# Chromosome territories reposition during DNA damage-repair response

**DOI:** 10.1186/gb-2013-14-12-r135

**Published:** 2013-12-13

**Authors:** Ishita S Mehta, Mugdha Kulashreshtha, Sandeep Chakraborty, Ullas Kolthur-Seetharam, Basuthkar J Rao

**Affiliations:** 1 Department of Biological Sciences, Tata Institute of Fundamental Research, Mumbai, Maharashtra 400005, India

## Abstract

**Background:**

Local higher-order chromatin structure, dynamics and composition of the DNA are known to determine double-strand break frequencies and the efficiency of repair. However, how DNA damage response affects the spatial organization of chromosome territories is still unexplored.

**Results:**

Our report investigates the effect of DNA damage on the spatial organization of chromosome territories within interphase nuclei of human cells. We show that DNA damage induces a large-scale spatial repositioning of chromosome territories that are relatively gene dense. This response is dose dependent, and involves territories moving from the nuclear interior to the periphery and vice versa. Furthermore, we have found that chromosome territory repositioning is contingent upon double-strand break recognition and damage sensing. Importantly, our results suggest that this is a reversible process where, following repair, chromosome territories re-occupy positions similar to those in undamaged control cells.

**Conclusions:**

Thus, our report for the first time highlights DNA damage-dependent spatial reorganization of whole chromosomes, which might be an integral aspect of cellular damage response.

## Background

DNA-damaging agents, both endogenous as well as exogenous, constantly pose a threat to the genome. Protecting the genome from damage and an efficient machinery to repair damage are essential for maintaining genomic integrity. A failure to do so can lead to mutations or result in cell death. Since, DNA is non-randomly and spatially distributed within nuclei, it is important to understand the interplay between DNA damage response (DDR) and nuclear or chromosome organization.

Genomes within interphase nuclei occupy discrete, three-dimensional regions known as chromosome territories (CTs) [1,2]. The non-randomness of CT organization within an interphase nucleus is conserved in organisms throughout evolution from flies to humans [1,3,4]. It has been speculated that the conserved arrangement of CTs is important for mediating genome functions [1,5-10].

Although non-random, changes in CTs have been associated with altered cellular physiology. Interestingly, the organization of interphase CTs has been shown to change during differentiation, quiescence and senescence [1,11-13] (Mehta IS, *et al*., unpublished work). Altered chromosome positioning has also been observed in some diseases and cancers [11,14,15]. Specifically, recent reports suggest that changes in whole chromosome or chromosomal domain positions are associated with modulations in transcriptional status [1,16-19] and chromatin architecture [1].

The link between transcription and CTs has been supported by studies where gene-rich chromosomes are present in the nuclear interior while gene-poor chromosomes are located at the periphery [20,21]. Further, studies have demonstrated that regions of DNA that are within the nuclear interior show higher transcriptional activity [22-24]. Although chromosome repositioning has been associated with varied cellular outputs, the underlying biological significance and the molecular mechanisms that mediate these relocalizations are poorly understood.

Local chromatin structure affects the damage frequency and repair mechanisms. It is interesting that open chromosomal material or chromatin incurs damage more easily than closed heterochromatin [25-29]. In addition, DNA composition is also known to impinge upon damage and repair mechanisms. DNA associated with features such as being at nuclear center, having higher gene density or GC content, or chromatin enriched with hyperacetylated histones, is more prone to damage [27,30,31]. Moreover, gene-rich regions and euchromatin are repaired faster than gene-poor and heterochromatic regions [25-29]. In addition to local changes in chromatin structure and functions [29,32], global alterations in chromosome architecture and position are likely to play an important role during DDR [33-38]. While studies have demonstrated that chromosome domains have to remain stationary following laser irradiation to assist repair, the studies focused on specific chromosomal loci and domains or only certain chromosomes [35,39-42]. However, in contrast, recent biophysical modelling studies have indicated that structural reorganization of genome and chromosome domains assists DDR [43,44]. Specifically, changes in the nuclear matrix attachment of chromosomes and repositioning of chromosome domains [45-49] have been hypothesized to assist cell cycle arrest, alter the transcription profile and the accessibility of damage sites to repair proteins [33,35,43]. Very recent reports for yeast have shown that damaged chromosomal domains undergo large-scale nuclear movements and it has been suggested that this phenomenon is vital for homology searches during homologous recombination (HR) [36,50-52]. Therefore, investigations of alterations in CTs, if any, are important for understanding the global effects of chromatin, chromosome packaging and nuclear architecture vis-à-vis DNA repair.

In this study, we have addressed the effect of DNA damage on CTs in primary human fibroblasts. We find that while most of the chromosomes do not change location, some gene-rich chromosomes, such as chromosomes 17, 19 and 20, reposition themselves after DNA damage in a dose- and time-dependent manner. Our observations also implicate the importance of DNA damage sensing in bringing about such alterations, whereby inhibiting the activity of DNA-dependent protein kinase (DNA-PKcs) and Ataxia Telangiectasia mutated kinase (ATM kinase) perturb this relocalization. The results also show that these spatial changes in CTs are reversible, since the DNA damage is repaired. Our report highlights an intricate relation between CTs and DNA damage response.

## Results and discussion

### Repositioning of select chromosomes after DNA damage

Corroborating earlier studies [21,52], we found that chromosomes maintain a non-random distribution with the gene-dense chromosomes 17, 19 and 20 distributed at the center of the nucleus and gene-poor chromosomes, such as 18, 21 and 22, distributed towards the nuclear periphery (Additional file 1, Additional file 2, Table 1).

**Table 1:** Positions of all human chromosomes in fibroblasts before and after DNA damage

**Chromosome number**	**Number of genes/megabases**	**Chromosome size (base pairs × 10**^**6**^**)**	**Chromosome position**	
			**Control**	**Treated**
19	25.38	63	Interior	Periphery
17	18.49	81	Interior	Periphery
20	14.34	63	Interior	Periphery
11	13.84	134	Periphery	Periphery
16	13.51	90	Intermediate	Intermediate
14	12.20	105	Intermediate	Intermediate
12	11.69	133	Periphery	Interior
1	11.28	245	Intermediate	Intermediate
15	10.68	100	Intermediate	Interior
7	9.33	158	Periphery	Periphery
X	9.21	152	Periphery	Periphery
9	8.94	134	Periphery	Periphery
6	8.94	170	Periphery	Periphery
10	8.10	135	Intermediate	Intermediate
2	7.68	243	Periphery	Periphery
8	7.65	145	Periphery	Periphery
21	7.49	46	Interior	Interior
3	7.40	199	Periphery	Periphery
5	7.08	181	Periphery	Periphery
18	6.84	77	Periphery	Periphery
Y	6.31	51	Interior	Interior
4	6.09	192	Periphery	Periphery
13	5.30	114	Periphery	Periphery
22	4.97	49	Interior	Interior

**Figure 1:**
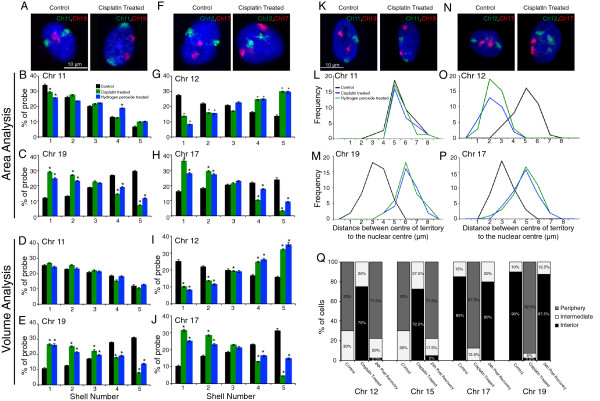
**Chromosome position in interphase nuclei of normal versus DNA-damaged fibroblasts.** CT positions of chromosomes 11, 12, 17 and 19 as assessed by 2D-FISH and 3D-FISHin response to treatment with 25 μM cisplatin, 1 mM H_2_O_2_ and 0.05% dimethyl sulphoxide (DMSO) (control). **(A, F)** Representative images for 2D-FISH. **(B, C, G, H)** Signal intensity histograms of 100 nuclei divided into five shells of equal area. **(D, E, I, J)** Signal intensity histograms of 100 nuclei divided into five shells of equal volume. Error bars represent SEM. * indicates *P* = 0.05. **(K, N)** Three-dimensional projections of 3D-FISH. **(L, M, O, P)** Frequency distribution of distance (μm) between geometric centers of the CT and the nucleus for at least 50 nuclei per sample. **(Q)** Frequency distribution of cells with CTs (12, 15, 17 and 19) positioned in the nuclear interior, intermediate and periphery before and after damage (cisplatin treatment), and post cisplatin wash-off. For all datasets, the frequency distribution for cisplatin-treated samples was statistically significantly different from control and 24-hour post cisplatin wash-off samples (*P* = 0.001), while no statistical difference was observed between control and 24-hour post cisplatin wash-off samples.

**Figure 2:**
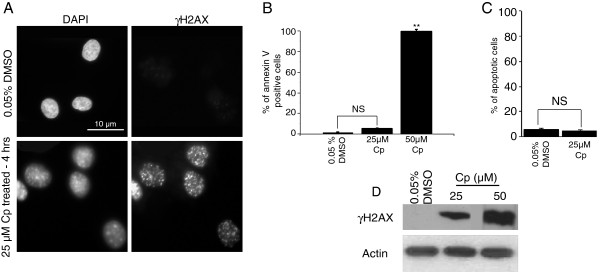
**DNA damage caused by cisplatin treatment.** Normal human dermal fibroblasts were treated with cisplatin and the extent of DNA damage and survival were monitored. Cells were treated for 4 hours with 25 μM cisplatin or 0.05% DMSO (control). γH2AX foci **(A)** and protein levels **(D)** increased in cisplatin-treated cells compared to their control counterparts. Annexin V staining **(B)** and FACS **(C)** were used to identify the percentage of cells undergoing apoptosis. ** indicates *P* = 0.001 NS: non-significant.

To investigate the relation between the spatial organizations of CTs and DDR, we analyzed the positions of all human chromosomes following DNA damage (Additional file 1, Additional file 2, Table 1). CT positioning was analyzed by 2D-FISH using IMACULAT methodology, which divides each nucleus into five shells of equal area (Figure 1, Additional file 1, Additional file 3) [53]. An extension of IMACULAT, where nuclei are divided into five shells of equal volume (Figure 1, Additional file 2, Additional file 3), and 3D-FISH analyses, were further used to corroborate the equal-area 2D-FISH analyses (Figure 1, Additional file 3). Using doses of DNA damaging agents that elicit DDR (Figure 2A,D) but do not induce apoptosis in cells (Figure 2B,C), we observed that most chromosomes (Additional file 1, Additional file 2 and Table 1) do not alter their localization within interphase nuclei following damage. However, interestingly a few chromosomes repositioned after DNA damage. Specifically, while chromosomes 17, 19 and 20 relocate from the nuclear interior to the periphery (Figure 1 and Additional file 4: Panel 1A,B,C,D), chromosomes 12 and 15 reposition towards the interior in a majority of cells (Figure 1 and Additional file 4). This relocation for chromosomes 12, 15, 17 and 19 is discernible in both equal-area and equal-volume partitioning (Figure 1, Additional file 1, Additional file 2 and Additional file 4: Panel 1A,B,C,D). The shift from an equal-area analysis to an equal-volume analysis will leave the outermost shell with less chromatin and the innermost shell with more chromatin, thereby dampening the slope (which is most prominent in control chromosome 15 in Additional file 1I compared to Additional file 2I and Additional file 4).

**Figure 3:**
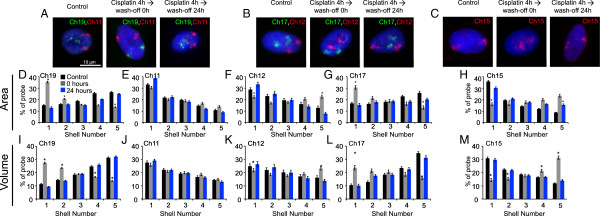
**Positions of chromosome territories in normal and DNA-damaged human lung fibroblasts.** The positions of chromosomes 11, 12, 15, 17 and 19 were delineated in normal human lung fibroblasts before and after DNA damage, and after 24 hours of recovery from damage. **(A, D, I, B, G, L)** As in normal human dermal fibroblasts, chromosomes 17 and 19 reposition from the nuclear interior to the periphery. **(B, F, K, C, H, M)** Chromosomes 12 and 15 relocate from the nuclear periphery to the interior after damage. On recovery, these chromosomes revert to their native locations after 24 hours (**A**, **B** and **C** and blue bars in panels **D**–**M**). Chromosome 11 does not show any relocation after DNA damage **(A, E and J)**. Scale bar: 10 μm. Error bars represent SEM. * indicates *P* = 0.05.

Earlier research demonstrated that the relative gene density of chromosomes plays a role in CT organization within cell nuclei. Interestingly, we observed a partial correlation between the relocation of CTs and their relative gene densities (Table 1) with some gene-rich chromosomes relocating following DNA damage (Table 1, Figure 1, Additional file 1 and Additional file 2). Conversely, none of the gene-poor chromosomes exhibited DNA-damage-induced CT relocation. This response was observed in normal human dermal fibroblasts (NHDFs) and normal human lung fibroblasts (NHLFs) (Figures 1 and 3, Additional file 5). Moreover, using DNA damaging agents that induced both single- and double-strand breaks (H_2_O_2_ and cisplatin), we observed that the same set of chromosomes displayed a ‘conserved’ repositioning pattern (Figure 1 and Additional file 4: Panel 1). We did not discern any changes in nuclear or CT volumes after DNA damage, thereby ruling out volume changes as a reason for CT repositioning following DDR (Additional file 4: Panel 1E,F).

Although, it is still far from clear why only certain chromosomes reposition after DNA damage, previous research by Falk and co-workers [29,54] clearly indicated an association between gene-rich chromosomes and the high frequency of phosphorylated histone 2A.X (γH2AX) foci. To assess if the frequency of damage affects DNA damage-dependent CT repositioning, we performed combined immuno-FISH experiments to analyze the occurrence of γH2AX foci on chromosomes that show relocalization after DNA damage. In concurrence with the previous research [29,54], we observed a slight increase in the number of γH2AX foci on gene-rich chromosomes that show relocation after DNA damage vis-à-vis the chromosomes that do not change location after DNA damage (Additional file 4: Panel 2). These results, suggest that chromosome relocalization after DNA damage might be an intrinsic component of DDR.

### Chromosome repositioning is DNA damage dose and time dependent

**Figure 4:**
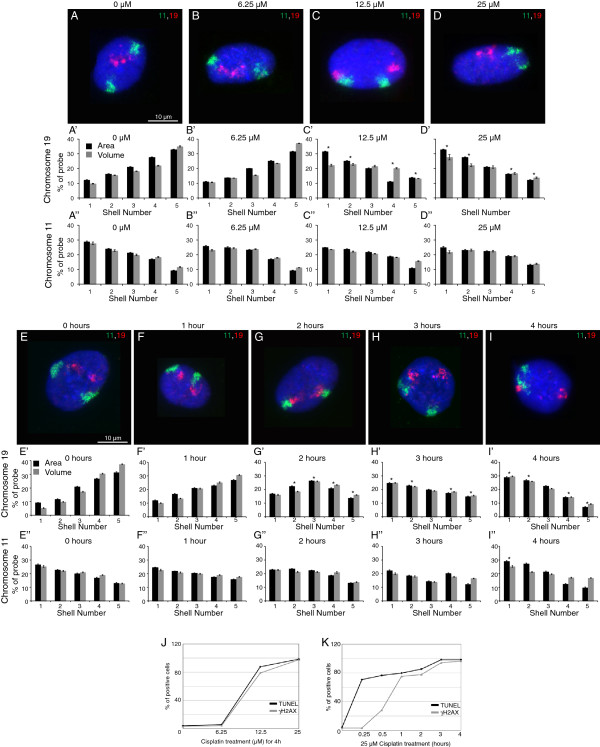
**Repositioning of chromosomes following DNA damage.** Cells were treated with varying doses of cisplatin (as indicated) for 4 hours. **(A, B, C, D)** 2D-FISH images. Histogram distributions for chromosomes 19 **(A’, B’, C’, D’)** and 11 **(A”, B”, C”, D”)**. Cells were treated with 25 μM cisplatin for varying time points (as indicated). **(E, F, G, H, I)** 2D-FISH images. Histogram distribution for chromosomes 19 **(E’, F’, G’, H’, I’)** and 11 **(E”, F”, G”, H”, I”)**. * indicates *P* = 0.05. **(J)** and **(K)**: TUNEL and γH2AX positive nuclei, under similar conditions. Scale bar: 10 μm.

To test whether the chromosome relocation is DNA damage dose dependent, cells were treated with varying doses of cisplatin for 4 hours. Chromosome 19 repositioned only when cells were treated with 12.5 μM or higher cisplatin concentrations, which was associated with an increase in double strand breaks (DSBs) [Terminal deoxynucleotidyl transferase mediated dUTP Nick End Labeling (TUNEL) and γH2AX foci] (Figure 4A,B,C,D,J). Chromosome 11 does not relocate and failed to show any repositioning for any dose of cisplatin (Figure 4A,B,C,D). The dose-dependent response indicates that below a certain threshold of DSBs, CT relocalization does not ensue.

Further, to assess whether chromosome relocation was linked to DSB recognition following breaks, we also analyzed the DSB kinetics (TUNEL assay and γH2AX). We observed that chromosome 19 relocation starts at about 2 hours following cisplatin treatment (Figure 4G) and coincides with an increasing γH2AX response (Additional file 4: Panel 2 and Figure 4K). This relocation is complete after 3 to 4 hours (Figure 4E,F,G,H,I). These findings were interesting because the repositioning was initiated only after DDR was elicited and thus, suggested that damage sensing and recognition preceded territory repositioning.

### Repositioning of chromosome territories is dependent upon DNA damage sensing

Since CT repositioning after DNA damage occurs post phosphorylation of H2AX, we hypothesized that factors involved in DSB recognition and downstream signaling might be involved. Specifically, we wanted to investigate the role of DSB sensors Ataxia Telangiectasia mutant (ATM)/Ataxia Telangiectasia and Rad3 related (ATR) and DNA Protein Kinase C (DNA-PKcs), which are involved in eliciting a repair response including cell cycle arrest, phosphorylation of H2AX and altering chromatin structure at the sites of damage [55-59].

**Figure 5:**
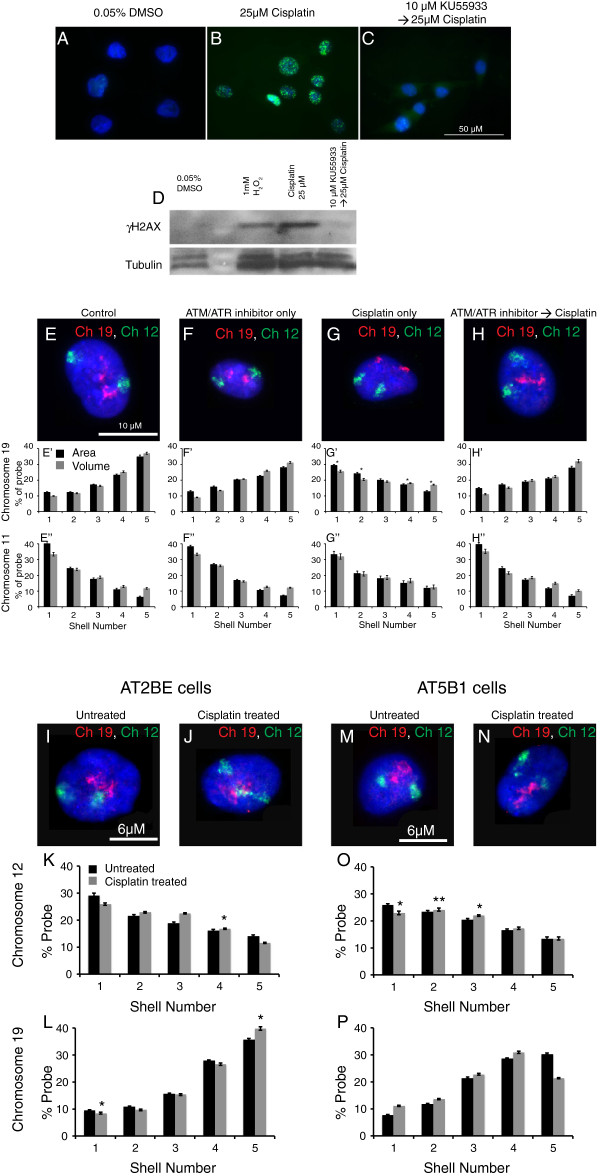
**Repositioning of chromosome territories following DNA damage requires ATM/ATR activity.** ATM activity in NHDFs was inhibited by treatment with 10 μM KU55933 for 1 hour. Pre-treatment with KU55933 inhibits phosphorylation of γH2AX after DNA damage as observed by IF staining **(A, B, C)** and Western blot analyses **(D)**. Chromosome 19 repositions from the nuclear interior **(E, E’, E”)** to the periphery after DNA damage **(G, G’, G”)**. This repositioning is inhibited in DNA-damaged cells pre-treated with ATM/ATR activity inhibitors **(H, H’, H”)**. Chromosome 11 remains at the nuclear periphery in cells subjected to the above treatments **(E–H, E’-H’, E”-H”)**. In ATM mutant fibroblasts AT2BE and AT5B1, chromosome 12 occupies the nuclear periphery **(I, J, K, M, N, O)** while chromosome 19 remains in the nuclear interior **(I, J, L, M, N, P)** in both undamaged cells (black bars) as well as in cells post DNA damage treatment (gray bars). * indicates *P* = 0.05 as assessed by ANOVA.

**Figure 6:**
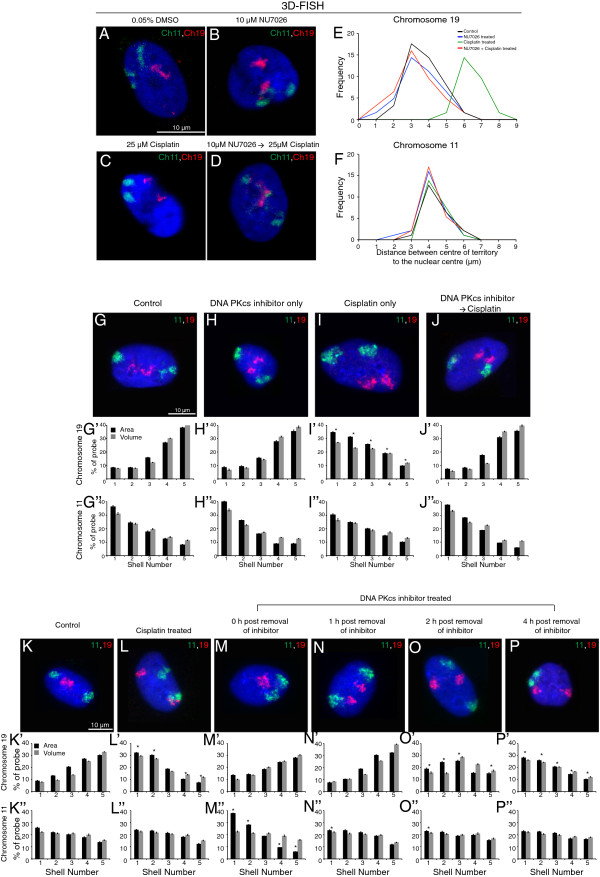
**Chromosome repositioning requires DNA-PKcs activity.** Positions of chromosomes 11 and 19 in control, only cisplatin, only DNA-PKcs inhibitor and inhibitor with cisplatin-treated cells as depicted by 3D-FISH **(A-F)** and 2D-FISH **(G-J)** analyses. Scale bar: 10 μm. Quantitation as in Figure 1. A delayed repositioning of chromosome 19 territories is observed in damaged cells after removal of the DNA-PKcs inhibitor from the culture medium **(K-P)** * indicates *P* = 0.05 as assessed by ANOVA.

Interestingly, cisplatin-treated cells did not reposition chromosomes in the presence of ATM/ATR inhibitors (Figure 5E,F,G,H). This result is consistent with our earlier observation with respect to the initiation of relocalization post γH2AX appearance (Figure 4 and Additional file 4: Panel 2). To further confirm these findings, we assessed the positions of chromosomes 11, 12 and 19 in fibroblasts that have non-functional ATM kinase, using AT2BE and AT5B1 cell lines derived from ataxia telangiectasia patients [60,61]. In concurrence with our inhibitor study, we observed that repositioning of chromosomes 12 and 19 did not occur after DNA damage in ATM mutant cell lines (Figure 5 and Additional file 6). This reiterates that functional ATM kinase is vital for DNA-damage-dependent CT repositioning. We observed that a similar loss of repositioning occurred when DNA-PKcs activity was inhibited (Figure 6 and Additional file 7). To validate this further, we altered the treatment regime so that the DNA-PKcs inhibitor was washed off midpoint during the 4 hours of cisplatin treatment. Notably, the ‘stalled’ chromosome 19 (which relocalizes in cells treated only with cisplatin) resumed repositioning when the DNA-PKcs inhibitor was removed (Figure 6). Thus, our study clearly indicates that DNA damage sensing might be required for relocation of CTs after DNA damage. Although the importance of DNA repair mechanisms in altering chromatin structure and functions [25,32] is known, this is possibly the first study to demonstrate that repair pathways not only influence chromosome behavior at specific domains or loci [34,43-48], but can also affect the whole CT distribution. Further, the results also highlight a potential crosstalk between DNA damage sensors and the spatial positioning of chromosomes.

### Chromosome territories revert back after repair

**Figure 7:**
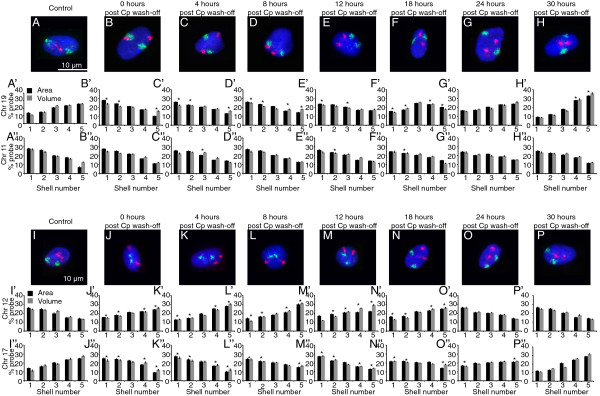
**Restoration of normal chromosome position occurs after washing off the DNA damage agent.** 2D-FISH data for cells treated with cisplatin (25 μM) for 4 hours and controls were collected at varying time periods after cisplatin wash-off (as indicated). Scale bar: 10 μm. The positions of chromosomes 11 and 19 **(A-H)** and 12 and 17 **(I-P)** were determined at each of these time points using 2D-FISH analyses.

**Figure 8:**
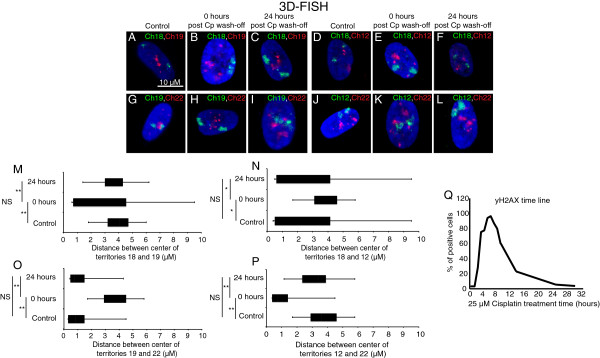
**Relocated chromosomes revert to distributions similar to control cells post cisplatin wash-off.** 3D-FISH analyses were performed to compute the pairwise distance distribution between relocating CTs 19 and 12 vis-à-vis static CTs 18 and 22 **(A-L)**. The pairwise distance distributions between CTs 19 and 18 **(A, B, C and M)**, 19 and 22 **(G, H, I and O)**, 12 and 18 **(D, E, F and N)**, and 12 and 22 **(J-L and P)** for at least 50 nuclei were measured. The box plots **(M, N, O, P)** span the second quartile, median and the fourth quartile of the pairwise distances, while negative and positive error bars represent the minimum and maximum distances. * indicates *P* = 0.01. **(Q)** Quantitation of γH2AX positive cells at various time points post cisplatin treatment.

We then wanted to investigate if the interplay between DNA damage sensing and CT relocalization was an important aspect of DDR. Since our treatments did not evoke a cell death response, we anticipated a reversion of CTs similar to their distribution in control cells post repair. Chromosomes 19 and 17, which showed peripheral distribution following damage, reverted to a more interior distribution like that in undamaged control cells, 18 to 24 hours post removal of the damaging agent (Figures 3 and 7A–H). The relocalization kinetics was also strikingly similar for chromosome 12, which reverted from an interior distribution following damage to a more peripheral distribution (Figures 3 and 7I-P) after cisplatin wash-off. To corroborate the reversal, the radial positions of relocating CTs (12, 15, 17 and 19) were determined with respect to static or non-relocating CTs (18 – peripheral and 22 – interior) (Additional file 8). Computing both the mean and distribution of inter-CT distances before and after damage, and after cisplatin wash-off indicated that the CTs reverted back to distributions like those in undamaged control cells, both in the interior and periphery of the nucleus (Figure 8, Additional file 8 and Additional file 9). The reversion of CTs to distributions similar to their normal undamaged counterparts was preceded by the disappearance of γH2AX foci (Figure 8Q, Additional file 3: Panel 2 and Additional file 9) [62,63]. It is important to note that both the initial CT response and the reversal followed the phosphorylation and dephosphorylation of H2AX, respectively. In addition to suggesting a link with chromatin components, our results also highlight a potential crosstalk between mechanisms that repair DNA and those that determine the location of chromosomal domains. It would be interesting to investigate the role of DNA repair factors in regulating chromosome territories and vice versa.

### Reversal of chromosome territories to locations similar to those of control cells post repair may require passage through mitosis

**Figure 9:**
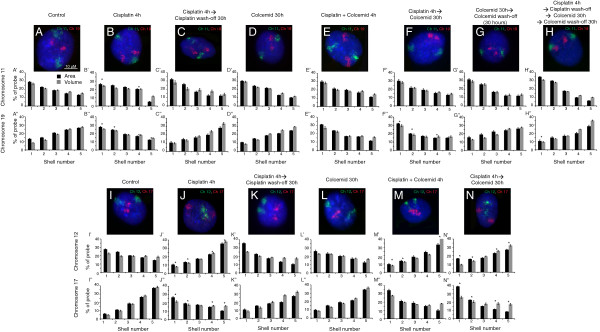
**Relocated chromosomes may require passage through mitosis to revert to distributions similar to control cells post cisplatin wash-off.** Normal DNA-damage-dependent repositioning **(A, B, I, J)** and repair-dependent reversal **(C, K)** of chromosomes 12 **(I, J, K)**, 17 **(I, J, K)** and 19 **(A, B, C)** in human dermal fibroblasts. In fibroblasts that were treated with cisplatin (25 μM) for 4 hours, and then allowed to repair in colchicine-containing media, the reversal of relocated chromosomes 12, 17 and 19 is not observed **(F, N)**. Colchicine, by itself, has no effect on the positions of CTs 12, 17 and 19 **(D, L)** and on the forward relocation of these CTs after DNA damage **(E, M)**. After removal of colchicine from the media, these relocated chromosomes revert back to locations similar to those of control cells **(G, H)**. Chromosome 11 territories remain at the nuclear periphery under all these conditions **(A-H)**. Scale bar: 10 μm.

Earlier studies suggested that the reversal of CTs to a distribution similar to control cells occurs post mitosis and requires nuclear rebuilding [12,64]. Therefore, we wanted to see if the reversal of CTs post DNA repair that we have observed here (Figures 7 and 8) also requires the cells to undergo mitotic exit. Moreover, since the DNA damage response leads to cell cycle arrest, CT reversal could occur within the same phase of the cell cycle. To test these two possibilities, we designed an experimental regime in which post cisplatin treatment (25 μM cisplatin for 4 hours), we incubated the cells in media containing colchicine (to cause mitotic arrest). We observed that colchicine-treated cells that had repositioned chromosomes after DNA damage failed to revert these CTs distributions as in control cells (Figure 9 and Additional file 10). Interestingly, the chromosomes did revert to distributions similar to their control counterparts within 30 hours post removal of the colchicine block (Figure 9H,H’,H” and Additional file 10). Moreover, the forward relocation of CTs following damage was not affected by colchicine’s action on the cells (Figure 9E,M). Although it appears that the forward relocation of CTs is microtubule independent, the reversal may still directly or indirectly require microtubules (as progression through mitosis is microtubule dependent). Thus, reversal of repositioned CTs to distributions similar to control cells post repair may require passage through mitosis, reinforcing "the nuclear rebuilding" hypothesis discussed above.

## Conclusions

This study reports a hitherto unknown association between DNA damage sensing and chromosome territories (CTs). We demonstrate that CT positions alter after DNA damage and are intrinsically coupled to the ability of cells to elicit a damage response dependent on ATM/ATR and DNA-PKcs activity. Local chromatin structure and DNA damage sensing mechanisms have been known to crosstalk with each other and mediate an efficient repair response [25-29,33]. Although the type and kinetics of repair are governed by the local chromatin structure (heterochromatin versus euchromatin) [25,29,32], the behavior of chromosomes as a whole has not been addressed thus far. In this regard, our results show reorganization of whole CTs in response to DNA damage. While some gene-rich CTs relocate (for example, 12 and 17) and others do not (for example, 11 and 14), a better correlation is observed in the lack of relocation of gene-poor chromosomes. It would be thus interesting to investigate further the properties of gene-rich CTs to determine their propensity to relocate.

Recent studies of yeast have hinted at the possibility of large-scale chromosome movement that ensue because of heightened mobility of broken DNA ends [29,35,36,49,50]. Chromatin repositioning, including the relocation of single or multiple chromosome loci or whole chromosomes, has been hypothesized to be a normal cellular response to radiation exposure [35-37,43,44]. Our findings not only support the hypothesis but also evidence the involvement of whole chromosomal mobility in DDR. These studies have also shown that the mobility of broken DNA ends influences HR efficiency. Although we cannot rule out such a possibility in our system (at least for the chromosomes that move to the interior), we do not hypothesize this to be a determining factor, since HR is not predominant in mammalian cells unlike yeast. Instead, we speculate that a potential bias in transcriptional activity and chromatin structure in these chromosomes might be relevant for such repositioning and needs to be addressed in the future. More importantly, we have addressed the relation between DNA damage response and nuclear organization vis-à-vis chromosomal localization.

Our results show that repositioned CTs revert to distributions similar to their control counterparts in a repair-dependent manner whereby restoration coincides with loss of γH2AX foci. Importantly, these raise fundamental questions about the link between DNA repair proteins and chromosomal relocalization, and suggest that CT reorganization could be an integral component of cellular DNA damage response. In conclusion, our report has investigated a novel aspect of DNA damage response and should lead to further studies aimed at understanding the role of nuclear complexity in maintaining genomic integrity.

## Materials and methods

### Cell culture and treatments

Early passage primary normal human dermal fibroblasts (NHDFs; Lonza) and normal human lung fibroblasts (Lonza) were cultured in 15% fetal bovine serum (FBS) supplemented DMEM and fibroblast growth medium (FGM) media supplemented with 2% FBS and growth supplements (Lonza). ATM mutant fibroblast lines AT2BE and AT5B1 (kindly provided by Dr Michael Weinfeld) were cultured in 10% FBS supplemented Dulbecco’s modified Eagle’s medium/nutrient mixture F-12 (D-MEM/F-12) (1:1 ratio). Cells were either treated with 1 mM H_2_O_2_ or with 25 μM cisplatin (Calbiochem) for 1.5 or 4 hours, respectively, unless mentioned otherwise in figure legends.

### Two-dimensional fluorescence *in situ* hybridization

2D-FISH was performed using the standard protocol [65]. Briefly, cells treated with hypotonic solution (0.075 M KCl) were fixed with 3:1 (v/v) methanol:acetic acid using the standard protocol. Cells dropped on slides were taken through an ethanol row followed by denaturation in 70% formamide at 70°C for 2 minutes. The slides were then taken through another ethanol row and air-dried at 37°C. Directly labelled total human chromosome DNA probes (Applied Spectral Imaging), denatured at 80°C for 7 minutes and re-annealed at 37°C for 20 minutes, were then applied to the denatured cells followed by hybridization for 18 hours. Post hybridization stringency washes were performed and then the cells were counter-stained with 4′,6-diamidino-2-phenylindole (DAPI) in Vectashield mounting medium.

At least 100 captured images (Axiovision software, Zeiss Axiovert 200 microscope) were run through IMACULAT [53], a methodology that divides each nucleus into five concentric shells of equal-area (Additional file 3). Since volume scales to the cube of the radius, a concentric equal-area partitioning of the nuclei biases the volume of the 3D shells towards the outermost shell, which has 29% of the total volume. To make the volumes equitable amongst shells, we enhanced the IMACULAT program to divide the nuclei into shells with areas proportional to 34, 20, 17, 16 and 13 (from the innermost shell to the outermost shell) (Additional file 3: Panel E and Additional file 11). The number of pixels of DAPI and the amount of the chromosome probe in these five shells were measured. Background normalization of the FISH signal was carried out using the mean pixel intensity within the segmented nucleus. The probe signal was normalized using following formula:

$$\frac{\%\;\mathrm{of}\;\mathrm{probe}\;\mathrm{in}\;\mathrm{shell}\;x÷\%\;\mathrm{of}\;\mathrm{DAPI}\;\mathrm{in}\;\mathrm{shell}\;x}{\%\;\mathrm{of}\;\mathrm{probe}\;\mathrm{in}\;\mathrm{all}\;\mathrm{shells}÷\%\;\mathrm{of}\;\mathrm{DAPI}\;\mathrm{in}\;\mathrm{all}\;\mathrm{shells}}$$

Histograms displaying these results and standard error bars representing the +/− SEM were plotted (Additional file 3B,C,D) [53]. One-way ANOVA and *t*-tests were performed.

### Three-dimensional fluorescence *in situ* hybridization

To conserve the three-dimensional structure of fibroblast nuclei, a previously described FISH methodology was used [65]. Cells, grown on slides, were fixed with 4% paraformaldehyde (PFA) and permeabilized using Triton X/saponin solution followed by four or five freeze-thaw cycles and depurination in 0.1 N HCl. Denaturation was performed using 70% and 50% formamide for precisely 3 and 1 minutes, respectively, at 73°C. Hybridization with processed probes (see 2D-FISH section) was carried out for 48 hours at 37°C. Post hybridization washes were carried out similar to 2D-FISH.

Stacks of 0.3-μm optical sections (with an average of eight) were captured using a Zeiss confocal laser-scanning microscope (LSM510). The distances between the geometric center of each chromosome territory and the nuclear center in at least 40 nuclei were measured (Bitplane Imaris software) (Additional file 3F) and frequency distribution curves were plotted. Simple statistical analyses were performed using the two-tailed Student’s *t*-test.

## Abbreviations

ATM: kinase Ataxia telangiectasia mutated kinase; ATR: Ataxia telangiectasia and Rad3 related; Ch: Chromosome; Cp: Cisplatin; CT: Chromosome territory; CT: Chromosome territory; DAPI: 4′,6-diamidino-2-phenylindole; DDR: DNA damage response; DMEM: Dulbecco’s modified Eagle’s medium; DMSO: Dimethylsulphoxide; DNA-PKcs: DNA-dependent protein kinase; DSB: Double stranded breaks; FACS: Fluorescence associated cell sorting; FBS: Fetal bovine serum; FGM: Fibroblast growth medium; HR: Homologous recombination; NHDF: Normal human dermal fibroblast; NHDF: Normal human dermal fibroblast; NHLF: Normal human lung fibroblast; NS: Not significant; PFA: Paraformaldehyde; SEM: Standard error of the mean; TUNEL: Terminal deoxynucleotidyl transferase dUTP nick end labeling; γH2AX: Phosphorylated histone 2A.X.

## Competing interests

The authors declare that they have no competing interests.

## Authors’ contributions

ISM conceptualized the project, carried out all the cellular biological experiments, the FISH assays, data collection, analysis and interpretation; participated in manuscript writing and provided the intellectual input. MK performed the experiments involving ATM mutants, participated in the discussions and helped in drafting the manuscript. SC devised all the computational tools for imaging, data analysis and interpretation and helped in finalizing the manuscript. UKS participated in data analysis and interpretation, provided scientific and critical intellectual input, and helped in drafting the manuscript. BJR conceptualized the project, participated in data analysis, interpretation and writing the manuscript and provided intellectual input. All authors read and approved the final manuscript.

## Supplementary Material

Additional file 1 **Position of chromosome territories before and after DNA damage.** Equal-area analysis: Cells were treated with 1 mM H_2_O_2_ for 90 minutes to induce DNA damage. Standard 2D-FISH assay was performed and at least 100 digital images were analyzed per chromosome by the IMACULAT equal-area algorithm. The graphs display the percentage amount of probe of each human chromosome in each of the eroded shells (*y*-axis) for control (black bars) and DNA-damaged (gray bars) fibroblasts, and the shell number on the *x*-axis. The standard error bars representing the standard errors of mean (SEM) were plotted for each shell for each graph. * indicates *P* = 0.05 as assessed by ANOVA. Click here for file

Additional file 2 **Position of chromosome territories before and after DNA damage.** Equal volume analysis: NHDFs were treated with 1 mM H_2_O_2_ for 90 minutes to induce DNA damage. A standard 2D-FISH assay was performed and at least 100 digital images were analyzed per chromosome by the IMACULAT equal-volume algorithm. The graphs display the percentage amount of probe of each human chromosome in each of the eroded shells (*y*-axis) for control (black bars) and DNA-damaged (gray bars) fibroblasts, and the shell number on the *x*-axis. The standard error bars representing the standard errors of mean (SEM) were plotted for each shell for each graph. * indicates *P* = 0.05 as assessed by ANOVA. Click here for file

Additional file 3 **2D- and 3D-FISH analysis for positioning chromosome territories.** NHDFs were probed with specific whole chromosome paints using 2D- or 3D-FISH. For 2D-FISH, images were taken and run through IMACULAT. The program divides each nucleus into five concentric shells of either equal area **(A)** or equal volume **(E)** and then measures the signal intensities of the probe and the amount of DNA in each shell. The amount of probe is then normalized with respect to the amount of DNA for each shell and histograms are plotted, which allow us to determine the positions of chromosomes as interior **(B)**, intermediate **(C)** or peripheral **(D)** within a cell nucleus. **(F)** Three-dimensional projections of 0.2-μm optical sections of nuclei subjected to 3D-FISH, imaged by confocal laser scanning microscopy and reconstructed using IMARIS software. The distance between the geometric centers of the chromosome territory and the nucleus was measured. Click here for file

Additional file 4: Panel 1 Chromosome positioning in control versus DNA-damaged cell nuclei. Control and 25 μM cisplatin-treated NHDFs were subjected to 2D-FISH to delineate the positions of chromosomes 15 and 20 before and after DNA damage. At least 100 images per sample were analyzed using standard 2D-FISH equal-area analysis. Chromosome 20 repositioned from the nuclear interior (black bars in **C**) to the periphery **(A, C)** while chromosome 15 relocated from the nuclear periphery (black bars in **D**) to the interior **(B, D)**, after treatment with 25 μM cisplatin (green bars in **C** and **D**) and 1 mM H_2_O_2_ (blue bars in **C** and **D**). No significant alterations are observed in the volumes of nuclei **(E)** or chromosome 11 and 19 CTs **(F)** before (black bars) or after treatment with 25 μM cisplatin (gray bars). Scale bar: 10 μm. * and # indicate *P* = 0.05 with respect to the control as assessed by ANOVA. **Panel 2**: Dynamics of γH2AX foci with respect to DNA-damage-dependent CT repositioning. The status of γH2AX foci and CT repositioning were analyzed using immuno-FISH analyses in undamaged cells, cells post cisplatin treatment (25 μM) for 4 hours (0 hours cisplatin wash-off) and then 24 hours post cisplatin wash-off. **(A, B, C, D, E, F)** Three-dimensional projections of immuno-FISH images. The number of γH2AX foci/nuclei was quantified for at least 50 nuclei per sample and is depicted in the box plot **(G)**. The error bars show the range (minimum and maximum) for the number of foci observed per nuclei. * indicates *P* = 0.05 with respect to the control as assessed by the standard Student’s *t*-test. **(H)** The number of foci per specific CT were also counted for at least 50 nuclei/sample using spot and surface algorithms from the IMARIS software. Click here for file

Additional file 5 Frequency distribution of cells with CTs positioned in the nuclear interior, intermediate and periphery before and after damage, and post recovery. Click here for file

Additional file 6: **DNA damage and chromosome positioning in cells from ataxia telangiectasia patients.** ATM mutant fibroblasts (AT2BE and AT5B1) were treated with cisplatin and the extent of DNA damage and survival were monitored. Cells were treated for 4 hours with 25 μM cisplatin or 0.05% DMSO (control). γH2AX foci **(A, C)** increased in cisplatin-treated cells compared to their control counterparts. Annexin V staining **(B, D)** was used to identify the percentage of cells undergoing apoptosis. The positions of chromosome 11 territories were determined in these fibroblasts before and after DNA damage **(E, F, G, H, I and J)**. Scale bar: 6 μm. * indicates *P* = 0.05 with respect to the control as assessed by ANOVA. Click here for file

Additional file 7 **Inhibition of DNA-PKcs activity.** Recruitment of DNA-PKcs foci that occurs after DNA damage (**A** was inhibited in cells where phosphorylation of this protein was perturbed using 10 μM NU7026. Scale bar: 30 μm. The amount of protein γH2AX also decreases in cells treated with NU7026 after DNA damage compared to untreated damaged cells **(B)**. Click here for file

Additional file 8 **Model showing the predicted outcomes if after repair chromosomes revert to similar locations as non-relocating chromosomes. (A, B)** The positions of relocating chromosomes 12 and 19 vis-à-vis static or non-relocating chromosomes 18 and 22 in a control sample, post DNA damage sample and a sample after the damaging agent has been washed off. Click here for file

Additional file 9 **Distances between relocating versus static CTs before and after damage, and post cisplatin wash-off.** Pairwise distance distribution between CTs 17 and 18 **(A)**, 15 and 18 **(B)**, 17 and 22 **(C)** and 15 and 22 **(D)** were measured in control and 25 μM cisplatin-treated cells and also post 24 hours of recovery. The box plots span the second quartile, median and the fourth quartile of the pairwise distances, while negative and positive error bars represent the minimum and maximum distances. Click here for file

Additional file 10 **Flow cytometry analysis for cells that are prevented from passage through mitosis.** NHLFs **(A)** were treated with cisplatin for 4 h. Post cisplatin wash-off, they were incubated in 0.05 μg/ml colchicine for 30 hours and analyzed using flow cytometry. Mitosis is blocked for these cells and hence there is a higher population of the G2/M phase of the cell cycle compared to a control sample **(B)**. When the cells are left in normal media for 30 hours after a further colchicine wash-off they resume cycling and the G2/M population decreases to 24% **(C)**. Click here for file

Additional file 11 **Extended experimental procedures.** P.S: Gray-scale images in all three channels and 3D stacks for Figures 2, 6, 8 and S3 can be found on the link below: http://www.tifr.res.in/~dbs/faculty/bjr/mehta/Genome_Biology_7297118161044271.zip. Click here for file
