# Supplementary figures and images for: Chromosome territories reposition during DNA damage-repair response
Source: Genome Biol. 2013 Dec 13;14(12):R135. doi: 10.1186/gb-2013-14-12-r135 (PMC4062845; doi:10.1186/gb-2013-14-12-r135)

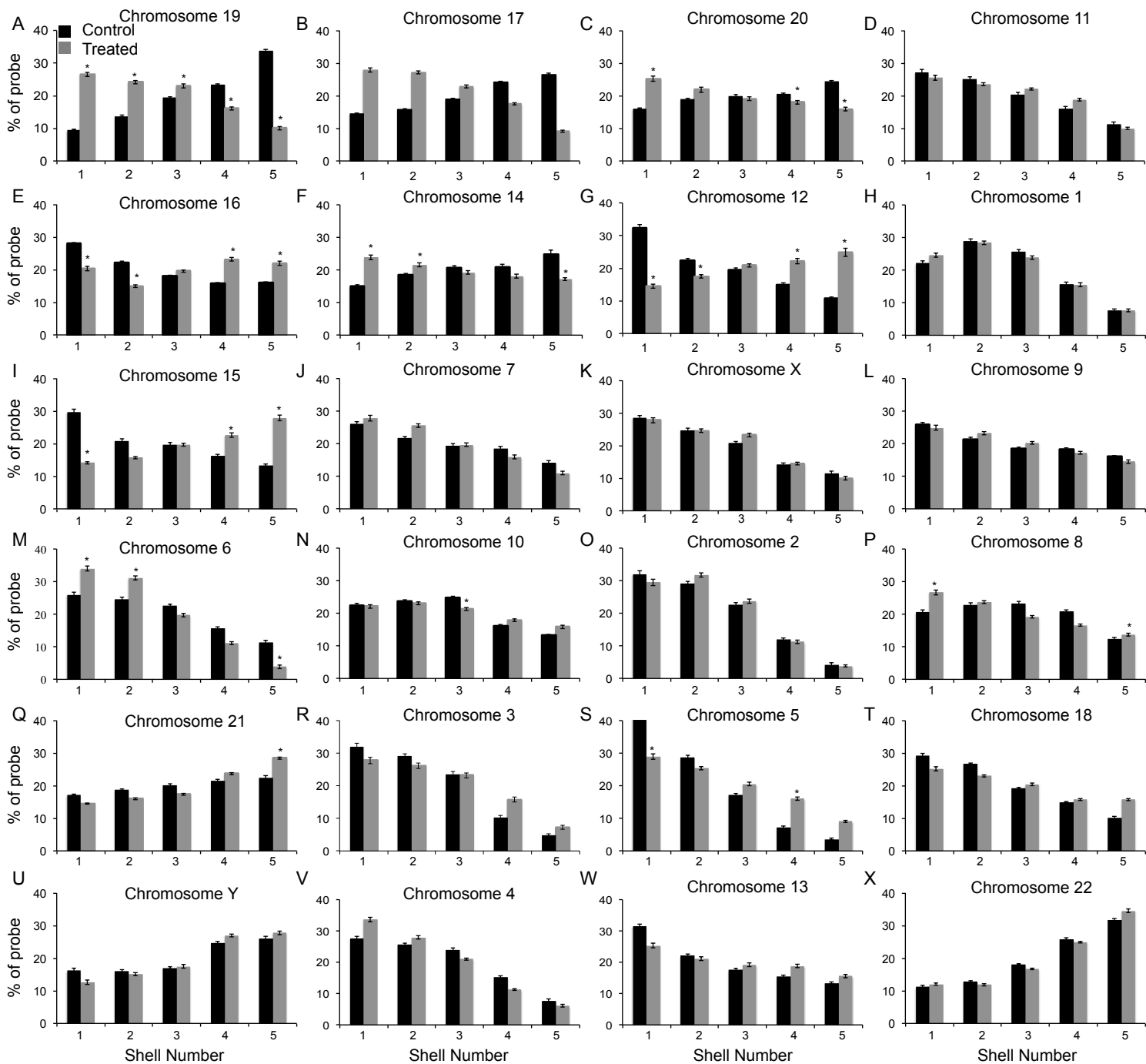

Supplement: Additional file 1 — Position of chromosome territories before and after DNA damage. Equal-area analysis: Cells were treated with 1 mM H2O2 for 90 minutes to induce DNA damage. Standard 2D-FISH assay was performed and at least 100 digital images were analyzed per chromosome by the IMACULAT equal-area algorithm. The graphs display the percentage amount of probe of each human chromosome in each of the eroded shells (y-axis) for control (black bars) and DNA-damaged (gray bars) fibroblasts, and the shell number on the x-axis. The standard error bars representing the standard errors of mean (SEM) were plotted for each shell for each graph. * indicates P = 0.05 as assessed by ANOVA. [file gb-2013-14-12-r135-S1.pdf]

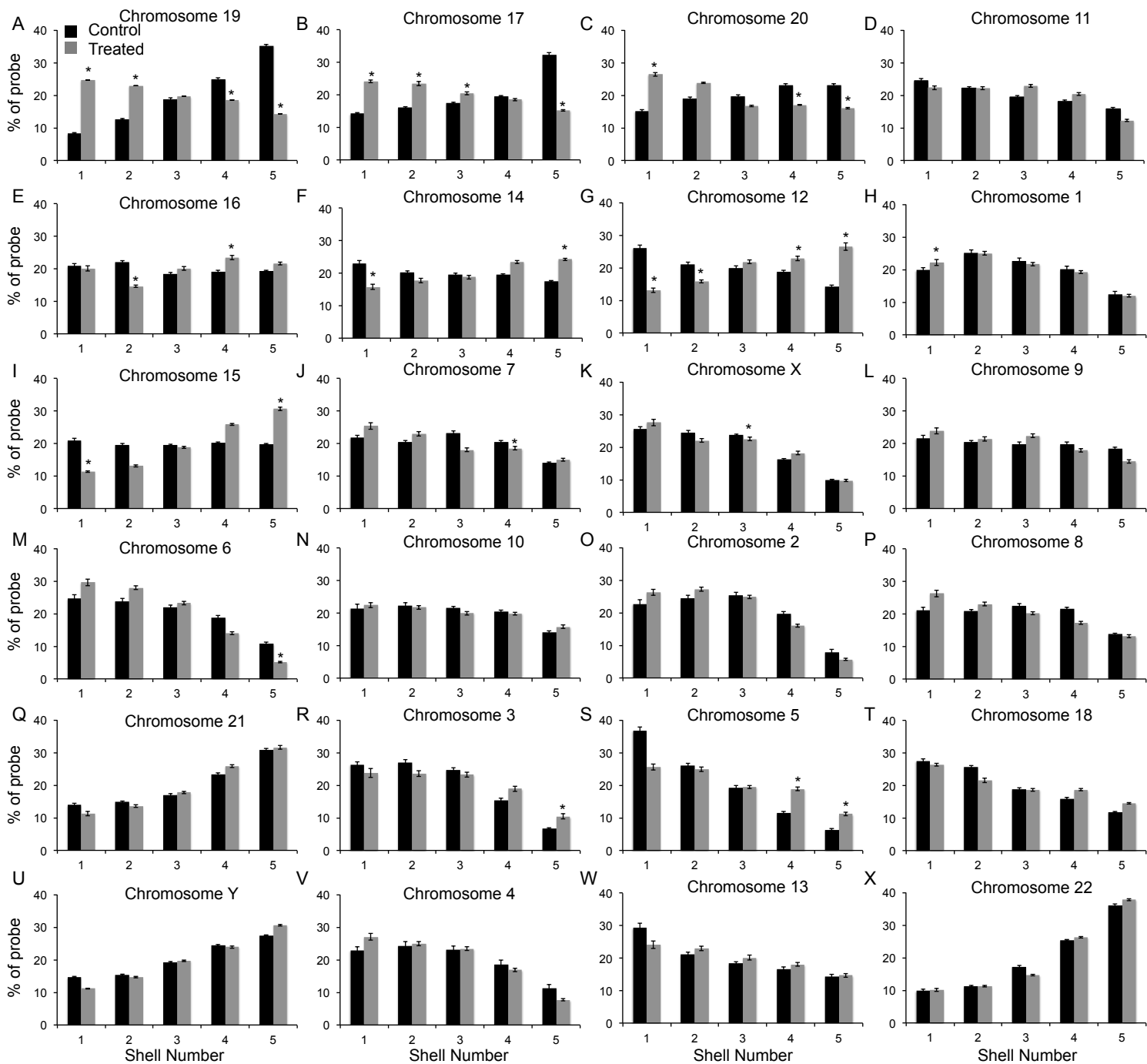

Supplement: Additional file 2 — Position of chromosome territories before and after DNA damage. Equal volume analysis: NHDFs were treated with 1 mM H2O2 for 90 minutes to induce DNA damage. A standard 2D-FISH assay was performed and at least 100 digital images were analyzed per chromosome by the IMACULAT equal-volume algorithm. The graphs display the percentage amount of probe of each human chromosome in each of the eroded shells (y-axis) for control (black bars) and DNA-damaged (gray bars) fibroblasts, and the shell number on the x-axis. The standard error bars representing the standard errors of mean (SEM) were plotted for each shell for each graph. * indicates P = 0.05 as assessed by ANOVA. [file gb-2013-14-12-r135-S2.pdf]

## Equal area analyses

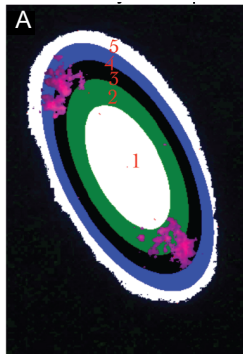

## 2D FISH analyses

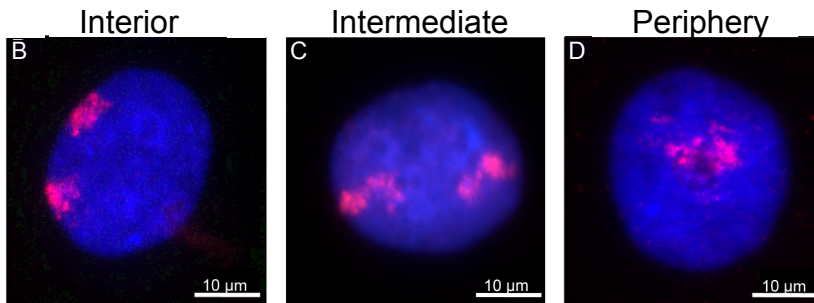

## 3D FISH analyses

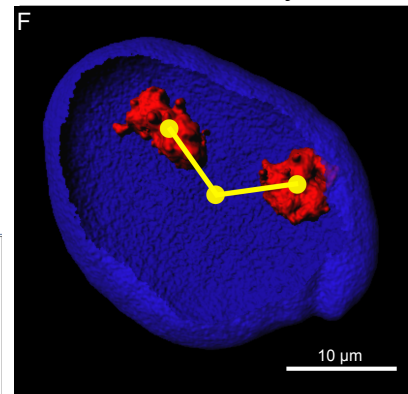

## Equal volume analyses

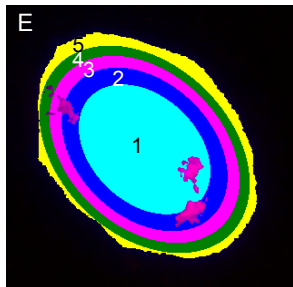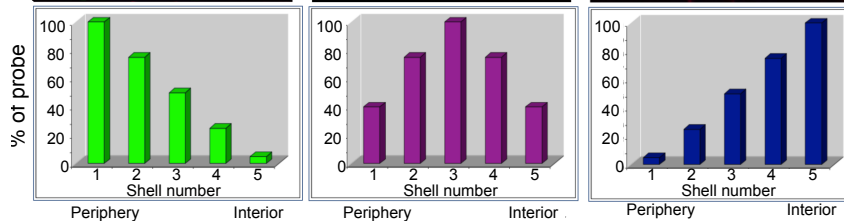

Supplement: Additional file 3 — 2D- and 3D-FISH analysis for positioning chromosome territories. NHDFs were probed with specific whole chromosome paints using 2D- or 3D-FISH. For 2D-FISH, images were taken and run through IMACULAT. The program divides each nucleus into five concentric shells of either equal area (A) or equal volume (E) and then measures the signal intensities of the probe and the amount of DNA in each shell. The amount of probe is then normalized with respect to the amount of DNA for each shell and histograms are plotted, which allow us to determine the positions of chromosomes as interior (B), intermediate (C) or peripheral (D) within a cell nucleus. (F) Three-dimensional projections of 0.2-μm optical sections of nuclei subjected to 3D-FISH, imaged by confocal laser scanning microscopy and reconstructed using IMARIS software. The distance between the geometric centers of the chromosome territory and the nucleus was measured. [file gb-2013-14-12-r135-S3.pdf]

## PANEL 1

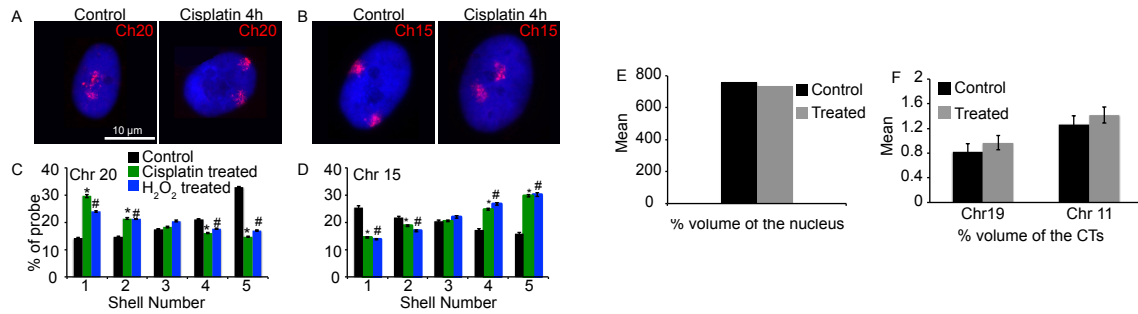

## PANEL 2

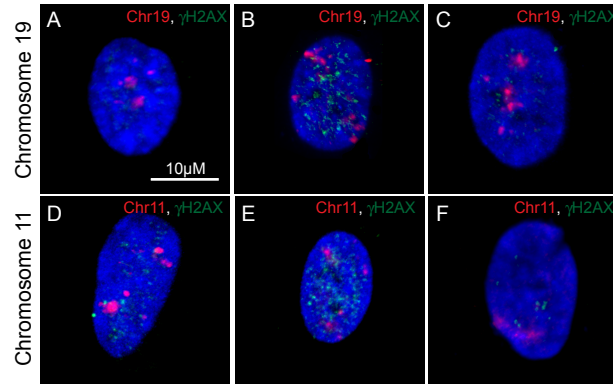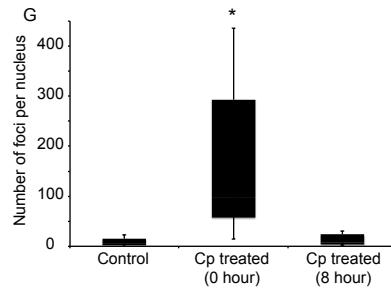

**H**

|            | Chr11     |        | Chr19     |        |
|------------|-----------|--------|-----------|--------|
|            | DSB [No.] | DSB/Mb | DSB [No.] | DSB/Mb |
| Control    | 0.64      | 0.005  | 0.48      | 0.008  |
| Cp treated | 14.08     | 0.105  | 9.44      | 0.150  |
| Recovery   | 2.28      | 0.017  | 1.20      | 0.019  |

Supplement: Additional file 4: Panel 1 — Chromosome positioning in control versus DNA-damaged cell nuclei. Control and 25 μM cisplatin-treated NHDFs were subjected to 2D-FISH to delineate the positions of chromosomes 15 and 20 before and after DNA damage. At least 100 images per sample were analyzed using standard 2D-FISH equal-area analysis. Chromosome 20 repositioned from the nuclear interior (black bars in C) to the periphery (A, C) while chromosome 15 relocated from the nuclear periphery (black bars in D) to the interior (B, D), after treatment with 25 μM cisplatin (green bars in C and D) and 1 mM H2O2 (blue bars in C and D). No significant alterations are observed in the volumes of nuclei (E) or chromosome 11 and 19 CTs (F) before (black bars) or after treatment with 25 μM cisplatin (gray bars). Scale bar: 10 μm. * and # indicate P = 0.05 with respect to the control as assessed by ANOVA. Panel 2: Dynamics of γH2AX foci with respect to DNA-damage-dependent CT repositioning. The status of γH2AX foci and CT repositioning were analyzed using immuno-FISH analyses in undamaged cells, cells post cisplatin treatment (25 μM) for 4 hours (0 hours cisplatin wash-off) and then 24 hours post cisplatin wash-off. (A, B, C, D, E, F) Three-dimensional projections of immuno-FISH images. The number of γH2AX foci/nuclei was quantified for at least 50 nuclei per sample and is depicted in the box plot (G). The error bars show the range (minimum and maximum) for the number of foci observed per nuclei. * indicates P = 0.05 with respect to the control as assessed by the standard Student’s t-test. (H) The number of foci per specific CT were also counted for at least 50 nuclei/sample using spot and surface algorithms from the IMARIS software. [file gb-2013-14-12-r135-S4.pdf]

# AT2BE cells

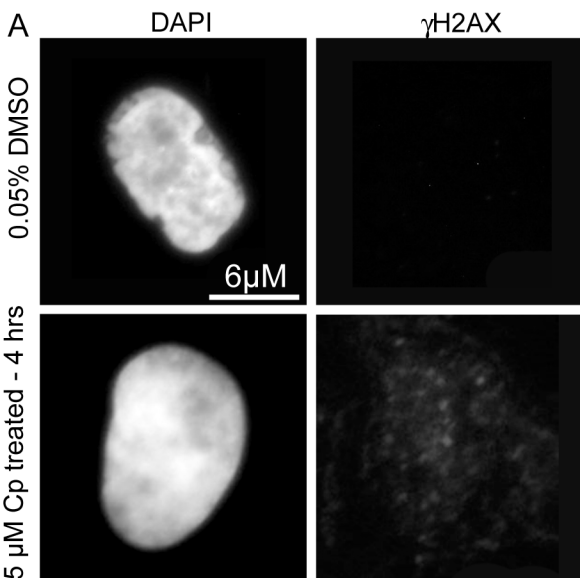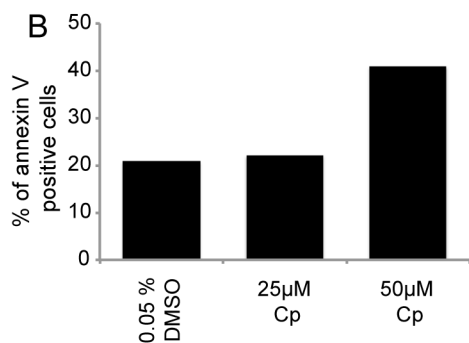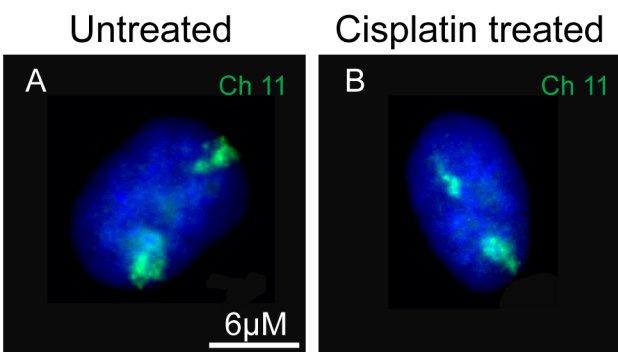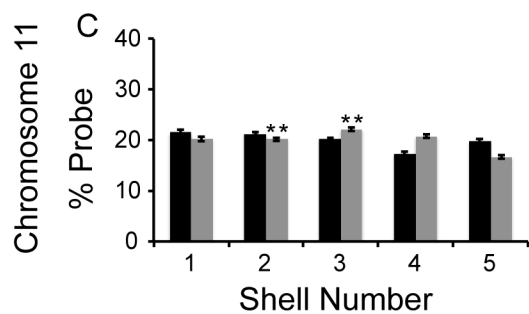

# AT5B1 cells

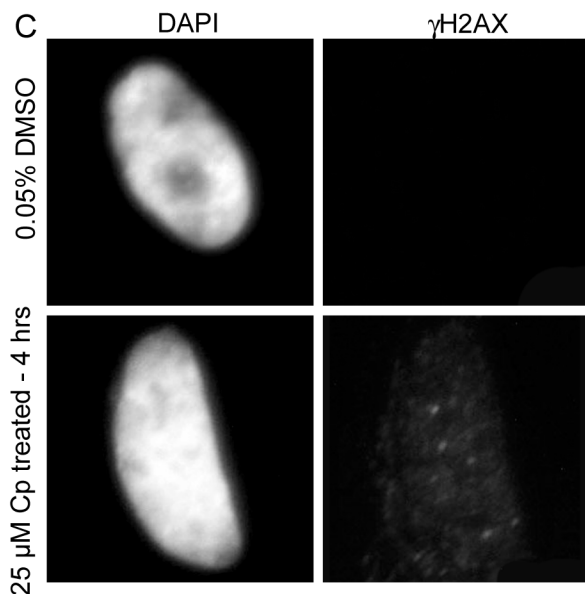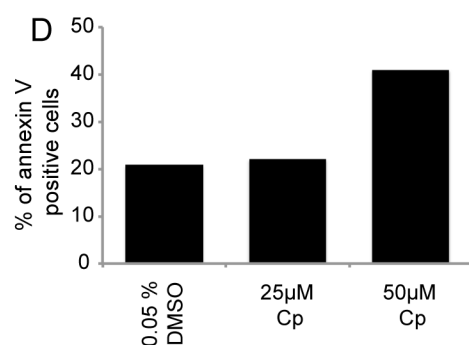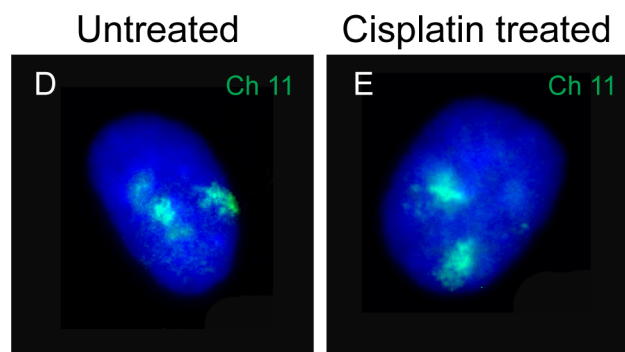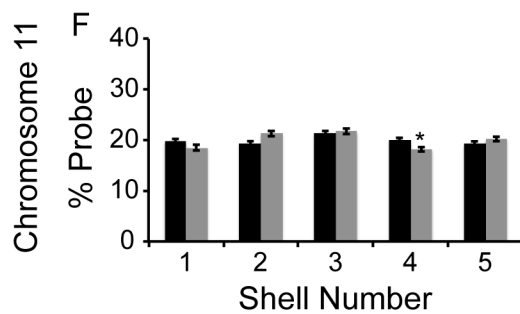

Supplement: Additional file 6: — DNA damage and chromosome positioning in cells from ataxia telangiectasia patients. ATM mutant fibroblasts (AT2BE and AT5B1) were treated with cisplatin and the extent of DNA damage and survival were monitored. Cells were treated for 4 hours with 25 μM cisplatin or 0.05% DMSO (control). γH2AX foci (A, C) increased in cisplatin-treated cells compared to their control counterparts. Annexin V staining (B, D) was used to identify the percentage of cells undergoing apoptosis. The positions of chromosome 11 territories were determined in these fibroblasts before and after DNA damage (E, F, G, H, I and J). Scale bar: 6 μm. * indicates P = 0.05 with respect to the control as assessed by ANOVA. [file gb-2013-14-12-r135-S6.pdf]

A

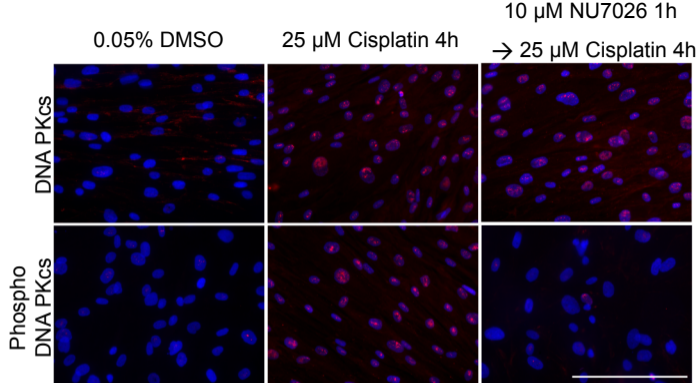

B

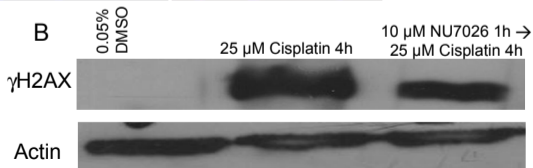

Supplement: Additional file 7 — Inhibition of DNA-PKcs activity. Recruitment of DNA-PKcs foci that occurs after DNA damage (A was inhibited in cells where phosphorylation of this protein was perturbed using 10 μM NU7026. Scale bar: 30 μm. The amount of protein γH2AX also decreases in cells treated with NU7026 after DNA damage compared to untreated damaged cells (B). [file gb-2013-14-12-r135-S7.pdf]

A

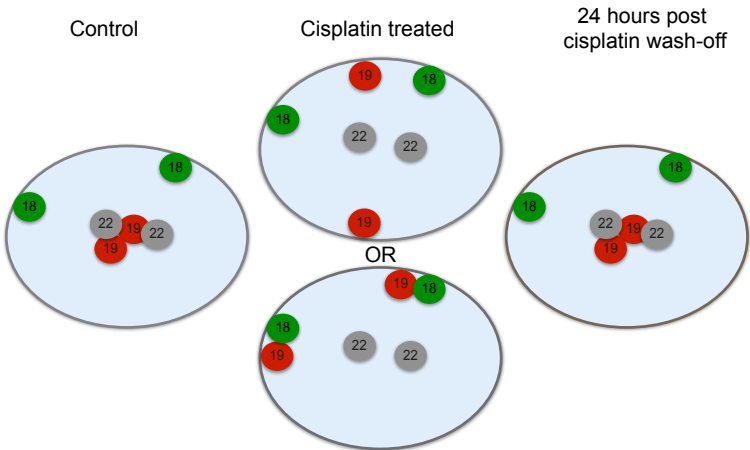

B

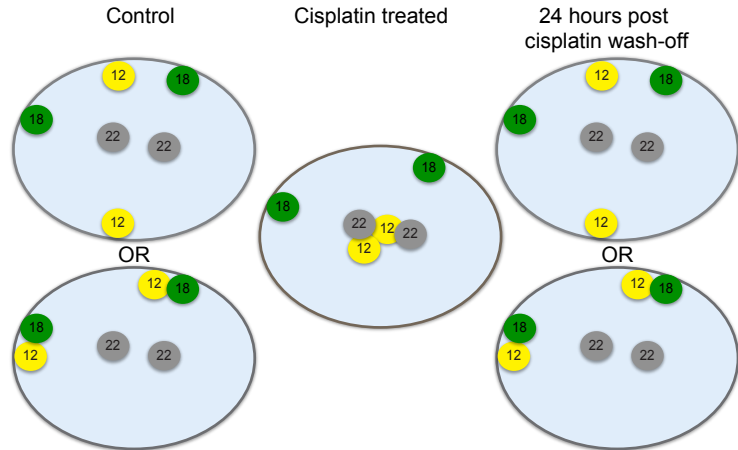

Supplement: Additional file 8 — Model showing the predicted outcomes if after repair chromosomes revert to similar locations as non-relocating chromosomes. (A, B) The positions of relocating chromosomes 12 and 19 vis-à-vis static or non-relocating chromosomes 18 and 22 in a control sample, post DNA damage sample and a sample after the damaging agent has been washed off. [file gb-2013-14-12-r135-S8.pdf]

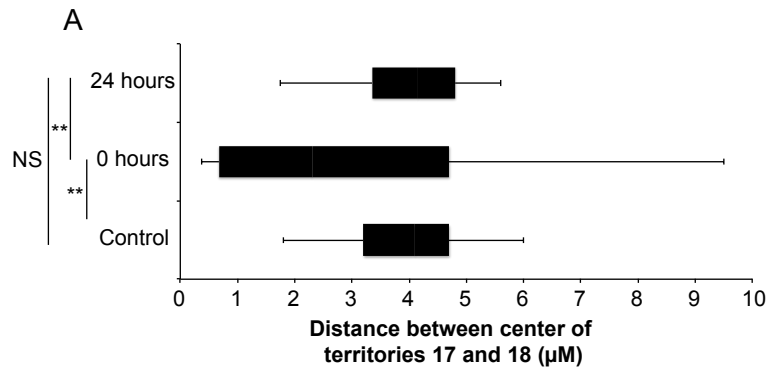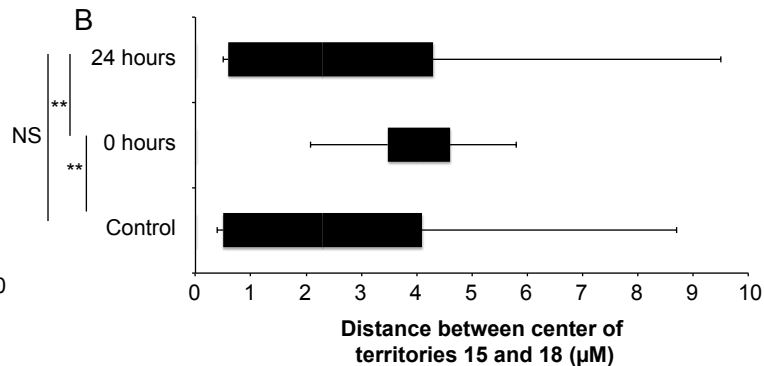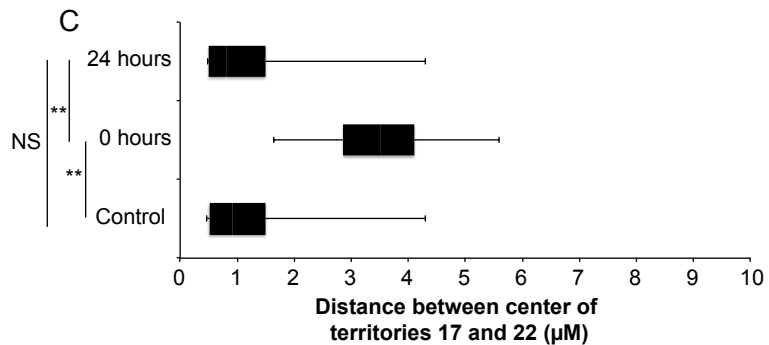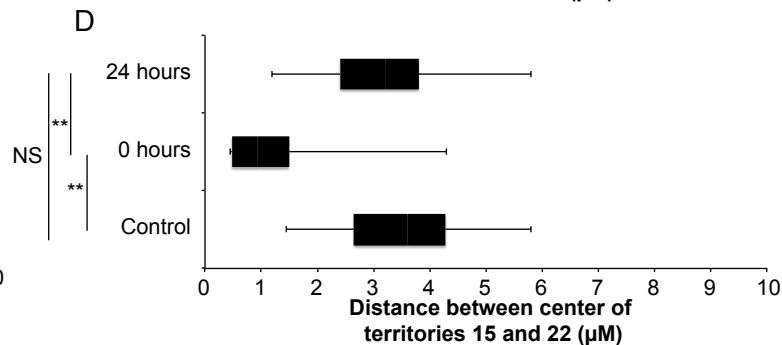

Supplement: Additional file 9 — Distances between relocating versus static CTs before and after damage, and post cisplatin wash-off. Pairwise distance distribution between CTs 17 and 18 (A), 15 and 18 (B), 17 and 22 (C) and 15 and 22 (D) were measured in control and 25 μM cisplatin-treated cells and also post 24 hours of recovery. The box plots span the second quartile, median and the fourth quartile of the pairwise distances, while negative and positive error bars represent the minimum and maximum distances. [file gb-2013-14-12-r135-S9.pdf]

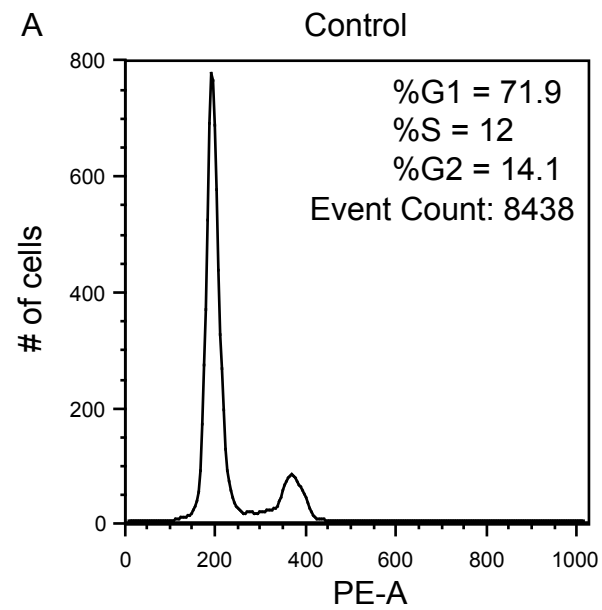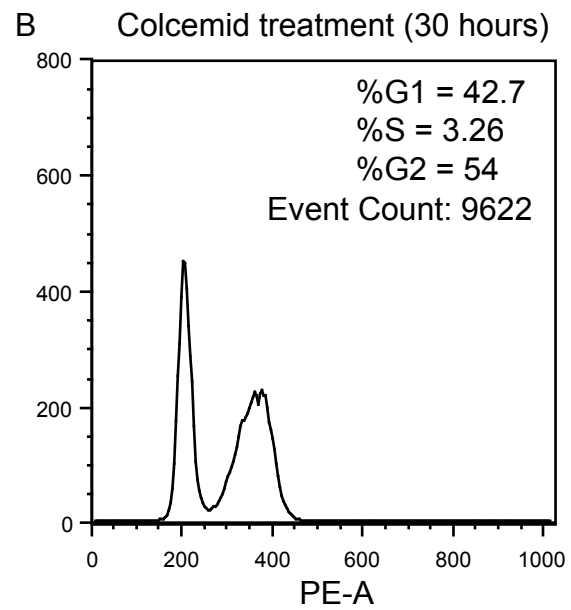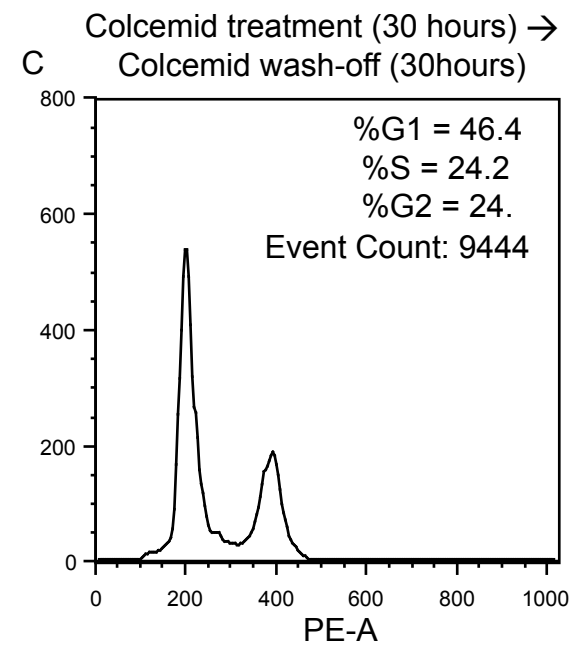

Supplement: Additional file 10 — Flow cytometry analysis for cells that are prevented from passage through mitosis. NHLFs (A) were treated with cisplatin for 4 h. Post cisplatin wash-off, they were incubated in 0.05 μg/ml colchicine for 30 hours and analyzed using flow cytometry. Mitosis is blocked for these cells and hence there is a higher population of the G2/M phase of the cell cycle compared to a control sample (B). When the cells are left in normal media for 30 hours after a further colchicine wash-off they resume cycling and the G2/M population decreases to 24% (C). [file gb-2013-14-12-r135-S10.pdf]
