# Supplementary material for: Chromosome territories reposition during DNA damage-repair response
Source: Genome Biol. 2013 Dec 13;14(12):R135. doi: 10.1186/gb-2013-14-12-r135 (PMC4062845; doi:10.1186/gb-2013-14-12-r135)
Supplement: Additional file 5 — Frequency distribution of cells with CTs positioned in the nuclear interior, intermediate and periphery before and after damage, and post recovery. [file gb-2013-14-12-r135-S5.pdf]

	Positions	Control	Cisplatin treated 4h	Post Cisplatin wash-off
**Chromosome 12**	**Interior**	0 ± 0	75 ± 8.67	2.5 ± 2.38
	**Intermediate**	30 ± 5.12	25 ± 5.03	20 ± 5.25
	**Peripheral**	70 ± 5.19	0 ± 0	77.5 ± 5
**Chromosome 15**	**Interior**	0 ± 0	72.5 ± 9.23	5 ± 4.56
	**Intermediate**	30 ± 5.89	27.5 ± 6.22	17.5 ± 4.92
	**Peripheral**	70 ± 5.17	0 ± 0	77.5 ± 5.98
**Chromosome 17**	**Interior**	85 ± 2.67	0 ± 0	80 ± 5.18
	**Intermediate**	15 ± 5.14	12.5 ± 4.06	20 ± 5.23
	**Peripheral**	0 ± 0	87.5 ± 3.56	0 ± 0
**Chromosome 19**	**Interior**	90 ± 2.35	2.5 ± 3.12	87.5 ± 2.12
	**Intermediate**	10 ± 3.12	5 ± 6.03	12.5 ± 4.12
	**Peripheral**	0 ± 0	92.5 ± 1.32	0 ± 0
