# Supplementary material for: Chromosome territories reposition during DNA damage-repair response
Source: Genome Biol. 2013 Dec 13;14(12):R135. doi: 10.1186/gb-2013-14-12-r135 (PMC4062845; doi:10.1186/gb-2013-14-12-r135)
Supplement: Additional file 11 — Extended experimental procedures. P.S: Gray-scale images in all three channels and 3D stacks for Figures 2, 6, 8 and S3 can be found on the link below: http://www.tifr.res.in/~dbs/faculty/bjr/mehta/Genome_Biology_7297118161044271.zip. [file gb-2013-14-12-r135-S11.docx]

**Additional file**

1. **Figure Legends**

**Supplementary Figure 1: Positions of chromosome territories before and after DNA damage – Equal area analysis:** Cells were treated with 1mM H_2_O_2_ for 90 minutes to induce DNA damage. Standard 2D-FISH assay was performed and at least 100 digital images were analysed per chromosome by the IMACULAT equal area algorithm. The graphs display the % probe of each human chromosome in each of the eroded shells (y-axis) for control (black bars) and DNA damaged (grey bars) fibroblasts, and the shell number on the x-axis. The standard error bars representing the standard errors of mean (SEM) were plotted for each shell for each graph. * indicates p-values of 0.05 respectively as assessed by ANOVA.

**Supplementary Figure 2: Positions of chromosome territories before and after DNA damage – Equal volume analysis:** NHDFs were treated with 1mM H_2_O_2_ for 90 minutes to induce DNA damage. Standard 2D-FISH assay was performed and at least 100 digital images were analysed per chromosome by the IMACULAT equal volume algorithm. The graphs display the % probe of each human chromosome in each of the eroded shells (y-axis) for control (black bars) and DNA damaged (grey bars) fibroblasts, and the shell number on the x-axis. The standard error bars representing the standard errors of mean (SEM) were plotted for each shell for each graph. * indicates p-values of 0.05 respectively as assessed by ANOVA.

**Supplementary Figure** **3: 2D and 3D-FISH analysis for positioning of chromosome territories:** NHDFs were probed with specific whole chromosome paints using 2D or 3D FISH. For 2D-FISH, images were then taken and run through IMACULAT. The program divides each nucleus into five concentric shells of either equal area (A) or equal volume (E) and then measures the signal intensities of probe and the amount of DNA in each shell. The amount of probe is then normalised with respect to the amount of DNA for each shell and then histograms are plotted which allow us to determine the positions of chromosomes in terms of Interior (B), Intermediate (C) and Peripheral (D) within a cell nucleus. 3D projections of 0.2µm optical sections of nuclei subjected to 3D-FISH imaged by confocal laser scanning microscopy and reconstructed using IMARIS software (F). The distance between the geometric centers of the chromosome territory and the nucleus was measured (F).

**Supplementary Figure 4 – Panel 1: Chromosome positioning in control v/s DNA damaged cell nuclei:** Control and 25µM cisplatin treated NHDFs were subjected to 2D-FISH for delineating the positions of chromosome 15 and 20 before and after DNA damage. At least 100 images per sample were analysed using standard 2D-FISH equal area analysis. Chromosome 20 repositioned from the nuclear interior (black bars in C) to the periphery (A, C) while chromosome 15 relocated from the nuclear periphery (black bars in D) to the interior (B, D), upon treatment with 25µM cisplatin (blue bars in C and D) and 1mM H_2_O_2_ (green bars in C and D). No significant alterations are observed in the volumes of nuclei (E) or chromosome 11 and 19 CTs (F) before (black bars) and after treatment with 25µM cisplatin (grey bars). Scale bar = 10µm. * and # indicate p-values of 0.05 with respect to the control as assessed by ANOVA. **Panel 2 -** **Dynamics of γH2AX foci with respect to DNA damage dependent CT repositioning:** The status of γH2AX foci and CT repositioning were analysed using immuno-FISH analyses in undamaged cells, cells post cisplatin treatment (25µM) for 4 hours (0 hours cisplatin wash-off) and then 24 hours post cisplatin wash-off. Panels A-F display 3D projections of immuno-FISH images. The number of γH2AX foci/nuclei was quantified in at least 50 nuclei per sample and is depicted in the box plot G. The error bars displays the range (minimum and maximum) of number of foci observed per nuclei. *indicate p-values of 0.05 with respect to the control as assessed by standard student’s t-test. Number of foci per specific CT were also counted for at least 50 nuclei / sample (panel H) using spot and surface algorithms from IMARIS software.

**Supplementary Figure 5: DNA damage and chromosome positioning in Ataxia telangiectasia patient cells:** ATM mutant fibroblasts (AT2BE and AT51B) were treated with cisplatin and the extent of DNA damage and effect survival were monitored. Cells were treated for 4 hours with 25 µM Cisplatin or 0.05% DMSO (control). γH2AX foci (A and C) increased in cisplatin treated cells as compared to their control counterparts. Annexin V staining (B and D) was performed to score for percentage of cells undergoing apoptosis. Positions of chromosome 11 territories were determined in these fibroblasts before and after DNA damage. Scale bar = 6 µM. *p-values of 0.05 with respect to the control as assessed by ANOVA.

**Supplementary Figure 6: Inhibition of DNA-PKcs activity:** Recruitment of DNA-PKcs foci that occurs upon DNA damage (A) was inhibited in cells where phosphorylation of this protein was perturbed using 10 µM NU7026 (A). Scale bar = 30 µM. Further, the amount of γH2AX protein also decreases in cells treated with NU7026 after DNA damage as compared to untreated damaged cells (B).

**Supplementary Figure 7: Model showing the experimental design and predicted outcomes for testing if upon repair chromosomes revert to similar locations with respect to non-relocating chromosomes:** Models suggesting the positions of relocating chromosomes 12 and 19 viz-à-viz static or non-relocating chromosomes 18 and 22 in control, post DNA damage and after wash-off of the damaging agent has been represented in A and B.

**Supplementary Figure 8: Distances between relocating v/s static CTs before and after damage, and post cisplatin wash-off:** Pairwise distance distribution between CTs 17 and 18 (A), 17 and 22 (C), 15 and 18 (B), and 15 and 22 (D) were measured in control and 25µM Cp treated cells and also post 24 hour recovery. The box plots span the 2nd quartile, median and the 4th quartile of the pair-wise distances, while negative and positive error bars represent the minimum and maximum distances.

**Supplementary Figure 9: Flow cytometry analysis for cells that are prevented from passage through mitosis:** NHLFs (A) were treated with cisplatin for 4h and post cisplatin wash-off were incubated in 0.05µg/ml colchicine for 30 hours before performing a flow cytometry analyses on the same. These cells are blocked in mitosis and hence higher cell population is observed in G2/M phase of the cell cycle as compared to control sample (B). Upon further wash-off of colchicine, when the cells are left in normal media for 30 hours, they resume cycling with % G2/M population decreasing to 24% (C).

**Table S1: Frequency distribution of cells with CTs positioned in the nuclear interior, intermediate and periphery before and after damage, and post recovery.**

P.S: Gray-scale images in all three channels and 3D stacks for figures 2, 6, 8 and S3 can be found on the link below:

<http://www.tifr.res.in/~dbs/faculty/bjr/mehta/Genome_Biology_7297118161044271.zip>

**B) Extended experimental procedures:**

**Indirect Immunofluorescence:** Cells fixed with 4% PFA followed by permeabilisation using 0.1% Triton X-100 were subjected to dual staining experiments, whereby cells were incubated with primary antibodies followed by secondary antibodies for 1 hour each at room temperature.

Primary antibodies: Rabbit γH2AX (Abcam); mouse γH2AX (Abcam); mouse p-DNA PKcs (Abcam) and goat DNA PKcs (Santacruz Biotechnology) were used at dilutions 1:1000, 1:1000, 1:100, 1:90 respectively in PBS/1%NCS (v/v).

Secondary antibodies: Goat anti-rabbit conjugated with FITC (Abcam), Goat anti-rabbit conjugated with FITC (Abcam), donkey anti-goat conjugated with FITC (Abcam) and goat anti-mouse conjugated with rhodamine (Abcam) were used at 1:500 dilution in PBS/1%NCS (v/v).

**Inhibitors:** NHDFs were treated with inhibitors for phosphorylation of DNA PKcs and ATM/ATR. In order to inhibit phosphorylation of ATM/ATR, cells were subjected to 10µM KU55933 (Calbiochem) for 1 hour. Cells were treated with 10µM NU7026 (Calbiochem) for 1 hour to inhibit the activity of DNA PKcs. For mitotic rebuilding experiments, cells were blocked from passage through mitosis by prolonged incubation in 0.05 µg/ml of colchicine (Karyomax).

**TUNEL Assay:** Cells fixed with 4% PFA and permeabilised using 0.2% Triton X-100, were subjected to TUNEL reaction (DeadEnd™ Fluorometric TUNEL kit, Promega).

**Annexin V staining:** NHDFs, equilibrated with 1X binding buffer were incubated with Annexin V FITC antibody (1:10 dilution) for 15 minutes (FITC Annexin V Apoptosis Detection Kit I, BD Phasmingen). The antibody is washed with 1X binding buffer and the cells were mounted in DAPI. Percentage of Annexin V positive cells was assessed using Zeiss fluorescence microscope.

**Western Blotting:** Control and cisplatin treated NHDFs lysed in RIPA (150mM NaCl, 1% NP40, 0.5% Sodium deoxycholate, 0.1% SDS, 50mM Tris pH 8, 1mM PMSF, 1X PIC) and protein amounts were quantified using Bradford’s reaction. 2X SDS sample buffer was then added to the lysate, which was then boiled at 100 ºC. Whole cell lysates were then resolved on 6% or 15% SDS-PAGE gels in 1X SDS-PAGE at 90V. Rainbow coloured molecular weight markers (wide range, Sigma-Aldrich) were used to detect protein size. Proteins, electrophoretically transferred onto nitrocellulose membrane (Amersham Hybond™-C Extra, Amersham Biosciences) and incubated in blocking solution (4% (w/v) dried milk powder (Marvel) in 1X transfer buffer) overnight at 4ºC were incubated with primary antibody for γH2AX (rabbit) (abcam) and anti-actin (rabbit) (Abcam) diluted 1:1000 and 1:5000 respectively. Following three washes in 1X TBS-Tween 20, membranes were incubated in secondary antibody (HRP labelled donkey anti Rabbit and donkey anti mouse; Bangalore geneie) both diluted 1:3000 for 1 hour at RT. Chemiluminiscent substrate kit (Roche) was used for antibody detection. Intensity of the bands were quantified using ImageJ

**FACS:** Cells were trypsinised and fixed in ice-cold 70% ethanol. Samples were stored at -20ºC for overnight or until further use. Just prior to performing the flow cytometry, ethanol was washed off from the cells using 38mM Na citrate solution. Cells were then stained using propidium iodide solution (69µM propidium iodide, 200 µg RNase A, 0.01% Triton X-100 in 38mM NaCitrate solution).

**Equations for volume analyses:**

Assuming the nucleus to be a sphere, whereby r_1_…r_5_ are the radii of the shells such that the shells have equal volumes; r_1_ being the radii of the innermost shell while r_5_ of the outermost (See figure below).

S1

S5

r_1_

r_2_

r_3_

r_4_

r_5_

S3

S2

S4

Now, the volume of the innermost shell is S1= $\frac{4}{3}\pi r_{1}^{3}$ and the volume of the next shell S2 = $\frac{4}{3}\pi r_{2}^{3}-\frac{4}{3}\pi r_{1}^{3}$.

Since S1 = S2, we have

$$\frac{4}{3}\pi r_{2}^{3}-\frac{4}{3}\pi r_{1}^{3}= \frac{4}{3}\pi r_{1}^{3}$$

$$\boldsymbol{\therefore}\boldsymbol{r}_{\boldsymbol{2}}^{\boldsymbol{3}}\boldsymbol{= 2}\boldsymbol{r}_{\boldsymbol{1}}^{\boldsymbol{3}}$$

Similarly S3 = S1,

$$\frac{4}{3}\pi r_{3}^{3}-\frac{4}{3}\pi r_{2}^{3}= \frac{4}{3}\pi r_{1}^{3}$$

$$r_{3}^{3}= r_{1}^{3}+ {2r}_{1}^{3}$$

$$\boldsymbol{\therefore r}_{\boldsymbol{3}}^{\boldsymbol{3}}\boldsymbol{=}{\boldsymbol{3}\boldsymbol{r}}_{\boldsymbol{1}}^{\boldsymbol{3}}$$

$$\boldsymbol{Similarly, r}_{\boldsymbol{4}}^{\boldsymbol{3}}\boldsymbol{=}{\boldsymbol{4}\boldsymbol{r}}_{\boldsymbol{1}}^{\boldsymbol{3}}$$

$$\boldsymbol{Similarly, r}_{\boldsymbol{5}}^{\boldsymbol{3}}\boldsymbol{=}{\boldsymbol{5}\boldsymbol{r}}_{\boldsymbol{1}}^{\boldsymbol{3}}$$

Expressing all radii in terms of the innermost radius r_1_,

$\boldsymbol{r}_{\boldsymbol{2}}\boldsymbol{=}\sqrt[\boldsymbol{3}]{\boldsymbol{2}}\boldsymbol{r}_{\boldsymbol{1}}\boldsymbol{=}\boldsymbol{K}_{\boldsymbol{1}}\boldsymbol{r}_{\boldsymbol{1}}\boldsymbol{\ldots\ldots\ldots. where}\boldsymbol{K}_{\boldsymbol{1}}\boldsymbol{=1.26}$ **…. (1)**

$$\boldsymbol{r}_{\boldsymbol{3}}\boldsymbol{=}\sqrt[\boldsymbol{3}]{\boldsymbol{3}}\boldsymbol{r}_{\boldsymbol{1}}\boldsymbol{=}\boldsymbol{K}_{\boldsymbol{2}}\boldsymbol{r}_{\boldsymbol{1}}\boldsymbol{\ldots\ldots\ldots. where}\boldsymbol{K}_{\boldsymbol{2}}\boldsymbol{=1.44\ldots. (2)}$$

$$\boldsymbol{r}_{\boldsymbol{4}}\boldsymbol{=}\sqrt[\boldsymbol{3}]{\boldsymbol{4}}\boldsymbol{r}_{\boldsymbol{1}}\boldsymbol{=}\boldsymbol{K}_{\boldsymbol{3}}\boldsymbol{r}_{\boldsymbol{1}}\boldsymbol{\ldots\ldots\ldots. where}\boldsymbol{K}_{\boldsymbol{3}}\boldsymbol{=1.59}$$

$$\boldsymbol{r}_{\boldsymbol{5}}\boldsymbol{=}\sqrt[\boldsymbol{3}]{\boldsymbol{5}}\boldsymbol{r}_{\boldsymbol{1}}\boldsymbol{=}\boldsymbol{K}_{\boldsymbol{4}}\boldsymbol{r}_{\boldsymbol{1}}\boldsymbol{\ldots\ldots\ldots. where}\boldsymbol{K}_{\boldsymbol{4}}\boldsymbol{=1.71}$$

In order to estimate the proportional area assigned to each shell, we need to express the total area A in terms of each intermediate areas A_5_ … A_1_.

Now A_5_ is the whole area of the nuclei $(A=\pi r_{5}^{2})$. Using r_1_ as an intermediate variable, we express A in terms of r_4_ (this is why we expressed all radii in terms of r1).

$$A=\pi r_{5}^{2}=\pi{(K_{4}r_{1})}^{2}=\pi\left( \frac{K_{4}^{2}}{K_{3}^{2}} \right)K_{3}^{2}r_{1}^{2}=\pi\left( \frac{2.92}{2.53} \right)r_{4}^{2}=1.15\left( \pi r_{4}^{2} \right)= 1.15A_{4}$$

$$\boldsymbol{\therefore}\boldsymbol{A}_{\boldsymbol{4}}\boldsymbol{= 0.87}\boldsymbol{A}$$

Similarly,

$$\boldsymbol{\therefore}\boldsymbol{A}_{\boldsymbol{3}}\boldsymbol{= 0.71}\boldsymbol{A}$$

$$\boldsymbol{\therefore}\boldsymbol{A}_{\boldsymbol{2}}\boldsymbol{= 0.54}\boldsymbol{A}$$

$$\boldsymbol{\therefore}\boldsymbol{A}_{\boldsymbol{1}}\boldsymbol{= 0.34}\boldsymbol{A}$$

Therefore, the nuclei have to be divided into areas proportional to 34, 20, 17, 16 and 13 in order to have shells of equal volumes.

**Equations for area analysis – determining volume bias due to an equal area partitioning**

Let us assume that r_1_…r_5_ are the radii of the shells of a sphere such that the shells have equal areas; r_1_ being the radii of the innermost shell while r_5_ of the outermost.

Now, the area of the innermost shell is S1= $\pi r_{1}^{2}$ and the area of the next shell S2 is $\pi r_{2}^{2}-\pi r_{1}^{2}$, which is equal to S1. Hence,

$$\pi r_{2}^{2}-\pi r_{1}^{2}=\pi r_{1}^{2}$$

$$\therefore\pi r_{2}^{2}=2\pi r_{1}^{2}$$

$$r_{2}=\sqrt{2}r_{1}\ldots\ldots\ldots\ldots(1)$$

Similarly for the shell S3,

$$\pi r_{3}^{2}-\pi r_{2}^{2}=\pi r_{1}^{2}$$

$$\therefore\pi r_{3}^{2}=3\pi r_{1}^{2}$$

$$r_{3}=\sqrt{3}r_{1}\ldots\ldots\ldots\ldots(2)$$

Similarly,

$$r_{4}=\sqrt{4}r_{1}\ldots\ldots\ldots\ldots(3)$$

$$r_{5}=\sqrt{5}r_{1}\ldots\ldots\ldots\ldots(4)$$

Now volume of the entire nucleus is $\frac{4}{3}\pi r_{5}^{3}$. Using equation 4, we can write

$$\frac{4}{3}\pi r_{5}^{3}=\frac{4}{3}\pi({\sqrt{5})}^{3}r_{1}^{3}\ldots\ldots\ldots\ldots(5)$$

Let $\frac{4}{3}\pi r_{1}^{3}=V_{1}$………. (6)

Replacing equation 6 in 5, we get

The volume of the entire nucleus in terms of V_1_

$\frac{4}{3}\pi r_{5}^{3}=({\sqrt{5})}^{3}V_{1}$=11.15$V_{1}\ldots\ldots\ldots\ldots(7)$

Similarly,

The volume of the nucleus till r_4_

$\frac{4}{3}\pi r_{4}^{3}=({2)}^{3}V_{1}$=8$V_{1}\ldots\ldots\ldots\ldots(8)$

The volume of the nucleus till r_3_

$\frac{4}{3}\pi r_{3}^{3}=({1.73)}^{3}V_{1}$=5.17$V_{1}$…………(9)

The volume of the nucleus till r_2_

$\frac{4}{3}\pi r_{2}^{3}=({1.41)}^{3}V_{1}$=2.8$V_{1}\ldots\ldots\ldots\ldots(10)$

Now, the 5^th^ shell is the volume of the entire nucleus – the volume of the nucleus till r_4_.

Using equations 7 and 8, we have

$$\therefore5th shell=\left( 11.15-8 \right)V_{1}=3.15V_{1}$$

Expressing this as a %,

$$\therefore\% 5th shell=\frac{3.15}{11.15}*100=28.3\%$$

Similarly the 4^th^ shell is the volume of the nucleus till r_4_ – the volume of the nucleus till r_3_,

Using equations 8 and 9, we have

$$\therefore\% 4th shell=\frac{2.83}{11.15}*100=25.4\%$$

Similarly,

$$\therefore\% 3rd shell=\frac{2.37}{11.15}*100=21.3\%$$

$$\therefore\% 2nd shell=\frac{1.8}{11.15}*100=16.4\%$$

$$\therefore\% 1st shell=\frac{1}{11.15}*100=8.9\%$$

As a sanity check, 28.3+25.4+21.3+16.4+8.9 = 100%.

Thus, we can see that the innermost shell is under-represented by almost 11% while the outermost shell is over-represented by 8% in an equal area analyses.
